# Prebiotic fiber blend supports growth and development and favorable digestive health in puppies

**DOI:** 10.3389/fvets.2024.1409394

**Published:** 2024-05-30

**Authors:** Allison P. McGrath, Laura A. Motsinger, John Brejda, Leslie Hancock

**Affiliations:** ^1^Hill’s Pet Nutrition, Inc., Topeka, KS, United States; ^2^Alpha Statistical Consulting, Lincoln, NE, United States

**Keywords:** dog, puppy, growth, prebiotic, gastrointestinal

## Abstract

**Introduction:**

A healthy gastrointestinal (GI) microbiome has been shown to be essential for proper nutrient absorption and metabolism, maintenance of intestinal epithelial integrity and osmolarity, gut immunomodulation, and overall health. One of the most effective ways to promote a healthy GI microbiome is through dietary interventions, such as the addition of prebiotics. Prebiotics are substrates that are selectively utilized by the host GI microbiome through fermentation to confer a health benefit. However, research on prebiotics in companion animals is limited, especially in growing animals. Thus, this study was conducted to assess the effects of a novel prebiotic fiber blend on key parameters related to intestinal health and growth in puppies.

**Methods:**

Twenty-two puppies at least 4 months of age but not older than 10 months were fed a commercially available dry food during a prefeed period, and then fed a similarly formulated test food with the addition of the prebiotic fiber blend for a minimum of 90 days. Serum and fecal samples were collected at the end of the prefeed period and throughout the test period.

**Results:**

Puppies fed the test food grew as expected for puppies of this age. Complete blood count and serum chemistry analyses were clinically normal for all animals. Fecal score increased linearly, fecal moisture decreased linearly, and pH exhibited a cubic trend throughout the study duration. There was a linear increase in short-chain fatty acids throughout the study, which is associated with favorable digestive and overall health. The inflammatory cytokine interleukin-7 decreased linearly and interleukin-18 trended towards linear decrease.

**Conclusion:**

This study showed that puppies continued to grow and develop normally, and experienced serum and stool characteristics indicative of improved GI health when fed a growth food fortified with a novel prebiotic fiber blend. Furthermore, these results contribute to the overall understanding of the effects of prebiotics on the GI health of growing companion animals.

## 1 Introduction

In recent years, the intricate interplay between nutrition, the gastrointestinal (GI) microbiome, and overall health has become a focal point of both human and veterinary research. Across species, previous work has shown that a healthy GI microbiome is essential for proper nutrient absorption and metabolism, maintenance of intestinal epithelial integrity and osmolarity, and gut immunomodulation [reviewed in Lyu et al. (1)]. In addition, the GI microbiome has been shown to affect the hypothalamic-pituitary-adrenal axis, by which it may impact the body as a whole (2). A healthy GI microbiome has been shown to be an important contributor to optimal nutrient absorption and metabolism and, as such, it is essential to the proper growth and development of companion animals.

According to the National Research Council, dogs reach adulthood at 12 months of age, but studies suggest that growth may continue beyond this point (3, 4). Therefore, adequate nutrition is critical during this period to support proper growth and development. Studies in both humans and animals have shown that the composition of the GI microbiome early in life may not only play a role in the development of certain acute conditions, such as diarrhea, but may also influence the risk of some chronic diseases in adulthood, such as obesity, metabolic disorders, allergies, neurologic dysfunction, and even cancer (5, 6). Thus, the establishment of a robust GI microbiome during the first year of life is an important consideration in raising healthy pets.

Probiotics, prebiotics, and postbiotics are dietary approaches that have demonstrated beneficial GI effects in a variety of species both *in vivo* and *in vitro* (7–10). Probiotics are live microorganisms that, when consumed in adequate amounts, produce beneficial metabolites. Postbiotics include these metabolites produced from gut microorganisms or inactive microbial cell components that can be isolated and consumed (10, 11). Prebiotics, which constitute the subject of this present study, are substrates selectively utilized by the host microbiome, producing metabolites that confer a health benefit, and include any substance that the GI microbiome can use for fermentation (12, 13). Some categories of prebiotics include fructans, oligosaccharides, resistant starch, and pectin (13). Although a majority of research to date has been performed using probiotics, investigations into prebiotics have been increasing in companion animals. In both cats and dogs, prebiotic complex carbohydrates have demonstrated positive effects on stool quality, digestive health, and the GI microbiome (7, 14–18). Studies in both humans and animals have found that carbohydrate fermentation by the GI microbiome leads to the increased production of metabolites such as saccharolytic short-chain fatty acids (SCFAs), which have been shown to provide anti-inflammatory and immunomodulatory activity, among other effects (19–21). Common dog food processing methods such as baking and extrusion have been shown to support increased SCFA production when compared to a minimally-processed control (22).

To help establish and maintain a healthy GI tract, a proprietary prebiotic blend was developed that provides both high- and low-solubility fibers and fiber-bound polyphenols. Selected fibers included in this prebiotic fiber blend are sourced from end-products produced by the human food industry, contributing to the global effort to reduce food waste (23). Previous studies in healthy adult dogs and those with chronic gastroenteritis/enteritis showed that this novel prebiotic blend positively affected stool quality, pH, SCFA production, and GI microbiome signatures and have also helped improve clinical signs of GI disease (24–27). In a study in adult cats with constipation or diarrhea, this prebiotic fiber blend rapidly and effectively resolved clinical symptoms (28). Studies investigating the effects of prebiotic fiber consumption in adult companion animals are limited, and this lack of knowledge is even greater in growing dogs. As such, the present study was undertaken to assess the effects of this novel prebiotic fiber blend on key parameters such as stool quality, moisture, pH, and SCFA content that are related to gastrointestinal health, as well as impacts on growth and development in puppies.

## 2 Methods

### 2.1 Animals

Both male and female puppies of any breed, including mixed breed, that were at least 4 months and at most 10 months of age at the start of the pre-feed period were eligible for participation. Puppies were included if they weighed at least 2 kg, were in good health with no prior disease conditions, and had no antibiotic interventions for at least 1 month before study enrollment. Exclusion criteria included being previously diagnosed with a chronic condition, including, but not limited to, gastrointestinal and renal diseases. Puppies were also excluded if they had a history of food allergy and/or poor eating behavior. Twenty-two puppies were selected: 18 beagles, 2 Cavalier King Charles spaniel/poodle mixes, and 2 Cavalier King Charles spaniel/Bichon Frise mixes. The study population consisted of 10 males and 12 females, with 5 males neutered, 5 males intact, and all 12 females intact upon start of the prefeed period. Ages ranged from 7 to 10 months old with a mean age of 8.9 ± 0.61 months, and a mean body weight of 7.5 ± 1.51 kg at the start of the prefeed period.

Puppies were removed from the study if they: (1) lost 15% of their body weight; (2) did not eat for 3 days; (3) were diagnosed with a new disease in which they would not benefit to remain on the study; (4) if the diagnosis of a disease necessitated a change in food (e.g., renal disease); or (5) would benefit from removal from the study for any reason, as reported by a veterinarian. No animals needed to be removed from the study.

All animals were pair-housed at and maintained by the Hill’s Pet Nutrition Center and treated in accordance with Hill’s Global Animal Welfare Policy. All puppies were allowed normal socialization, including daily interaction with animal care technicians and other dogs, and enrichment activities such as daily access to toys, open courtyards, and a park. The design of the study did not interfere with the animals’ normal daily routine.

This study was performed with approval from the Hill’s Pet Nutrition Institutional Animal Care and Use Committee (IACUC) and in accordance with Hill’s Global Animal Welfare Policy. At no time were the dogs subjected to any procedures expected to cause pain or distress.

### 2.2 Diets

Puppies were fed once a day throughout both the prefeed and treatment periods. During the prefeed period, puppies were fed a commercially available dry food formulated to be complete and balanced for growing puppies. The food fed during the testing period was a similar nutrient composition to the prefeed food, but included an addition of a novel prebiotic fiber blend. The prebiotic fiber blend consisted of ground pecan shells, flaxseed, dried beet pulp, dried citrus pulp, and pressed cranberries. Table 1 presents the key nutrient levels of both the prefeed and test foods. Both the prefeed and test foods met the Association of American Feed Control Officials (AAFCO) guidelines for complete and balanced foods for growing dogs (29).

**Table 1:** Selected nutrient composition of the prefeed food^a^ and test food^b^ on a dry matter basis.

Nutrient	Value	
	Prefeed food	Test food
Calories (Atwater), kcal/kg	4104.90	4073.09
Protein (crude), %	29.82	27.71
Fat (crude), %	19.14	19.00
Fiber (crude), %	1.67	2.47
Total dietary fiber, %	8.06	8.93
Soluble fiber, %	1.92	1.18
Insoluble fiber, %	5.86	7.58
Methionine + cysteine, %	1.07	1.05
Phenylalanine + tyrosine, %	2.27	1.93
Arginine, %	1.70	1.87
Histidine, %	0.66	0.66
Isoleucine, %	1.05	1.08
Leucine, %	2.69	1.94
Lysine, %	1.50	1.74
Threonine, %	1.09	1.24
Tryptophan, %	0.28	0.35
Valine, %	1.28	1.28
Taurine, ppm	1169.37	1199.92
Calcium, %	1.62	1.54
Phosphorous, %	1.21	1.25
Potassium, %	0.86	0.90
Sodium, %	0.53	0.50

ppm, parts per million.

a Science Diet Puppy Original.

b Science Diet Puppy Chicken Brown Rice AB+.

### 2.3 Study design

Each puppy was fed the prefeed food during a 14-day prefeed period. After completion of the prefeed period, puppies were transitioned onto the test food containing the fiber blend. This transition was considered the start of the testing period, which continued for a minimum of 90 days. Puppies remained on the study until they reached 14 months of age (92 to 175 days). The animals were fed once daily, and water was available *ad libitum*.

### 2.4 Assessments

#### 2.4.1 Serum samples

Serum samples were collected after dogs were fasted for at least 12 h at the end of the prefeed period, on day 92 of consuming the test food, and upon study completion (14 months of age). Whole blood was analyzed for complete blood count (CBC) immediately after collection (Sysmex XN 1000-V, Sysmex America, Inc., Lincolnshire, IL, United States). Serum chemistry was analyzed within 24 h of collection (Cobas c501, Roche Diagnostics, Indianapolis, IN, United States) and then frozen at −80°C until needed for further analysis. Inflammatory cytokines were analyzed using a 13-plex canine cytokine/chemokine immunology multiplex assay (MILLIPLEX^®^ Canine Cytokine/Chemokine Magnetic Bead Panel, MilliporeSigma, Burlington, MA, United States). Any measurements for cytokines that were not detected, and thus less than the lowest level of detection, were replaced with the value of one half of the lowest level of detection for the purpose of these analyses. Serum IgA was analyzed by MLM Medical Labs, Inc. (Oakdale, MN, United States). Analytical methods and data from metabolomics will be presented in a separate publication.

#### 2.4.2 Fecal samples

Fecal samples were collected during the prefeed period, every other week for the first 60 days of the study, and monthly after day 60 until study completion. Whole feces were collected within 30 min of defecation. Fecal score was assessed immediately after collection, using a 1–5 scale, where grade 1 indicated stool with no solid form and >70% liquid and grade 5 meant stool was well-formed, cylindrically shaped and >80% firm. Fecal samples were then homogenized until visually uniform with pH and moisture measured immediately after homogenization, snap-frozen in liquid nitrogen, and stored at −70°C until needed for further analysis.

Additional analyses of fecal matter included ammonia, calprotectin, IgA, metabolomics, microbiome, and SCFA composition. Ammonia in feces was analyzed by the indophenol method. Fecal SCFAs were analyzed by Metabolon, Inc. (Morrisville, NC, United States). Fecal calprotectin and IgA were analyzed by MLM Medical Labs, Inc. (Oakdale, MN, United States). Metabolomics and microbiome analytical methods and data will be presented in a separate publication.

Weekly body weight and daily food intake were also measured and continuously monitored.

### 2.5 Statistical analyses

The experimental design was a longitudinal study in which puppies were fed a growth food for up to 175 days until the animals reached 14 months of age (the treatment period). Fecal scores and chemistry, short-chain fatty acids, and ln-transformed serum cytokines data were analyzed using a linear mixed-model with Day as the only fixed-effect in the model. An appropriate variance-covariance structure was fit to the data for each response variable to account for the correlation between the repeated measurements. The Akaike information criterion corrected fit statistic was used to select the best covariance structure for each response variable. The NOBOUND option was used to allow for negative variance component estimates. The Kenward-Roger adjustment (DDFM = KR) was used to estimate the denominator degrees-of-freedom in the *F*-tests.

Orthogonal polynomial contrasts were used to identify clinically meaningful trends over time. The time points in which the measurements were taken were unevenly spaced. Therefore, the orthogonal polynomial coefficients for the trends analysis were calculated using the MANOVA option in PROC GLM in SAS^®^ (SAS Institute Inc., Cary, NC). The last prefeed measurement was defined as Day 0 in the test period for the assessment of trends over time.

If clinically meaningful trends were identified, random coefficients models were fitted to the data to generate regression models to describe the trends (30, 31). Random intercepts and slope coefficients were included in the preliminary model, and the COVTEST option was used to determine if the variance components associated with these terms accounted for a significant amount of variation. For most analytes the variance component associated with random slopes was statistically not significant and dropped from the final model. The final model typically contained a fixed intercept and slope term, and a random intercept term.

Hypothesis testing was not performed on the safety labs (serum chemistry and CBC). Rather, summary statistics (*n*, mean, standard deviation, minimum, median and maximum) were reported for each lab parameter at each time point. In addition, change-from-baseline summary statistics were calculated for each lab parameter at each test period time point, with the last prefeed assessment used as baseline. All lab values that were outside their respective reference range were listed for review by a clinical veterinarian for clinical significance. The linear mixed-model analysis and random coefficient analysis was performed using PROC GLIMMIX in SAS^®^, version 9.4 (SAS Institute Inc., Cary, NC). The summary statistics for the safety labs were calculated using PROC MEANS in SAS^®^, version 9.4 (SAS Institute Inc., Cary, NC).

## 3 Results

### 3.1 Animals

The study was conducted between July 2022 and January 2023, enrolling 22 puppies. None were prematurely removed from the study. A limited number of adverse events were reported; all of which were only 1–2 days in duration and unrelated to the study food.

### 3.2 Food intake and body weight

**Figure 1:**
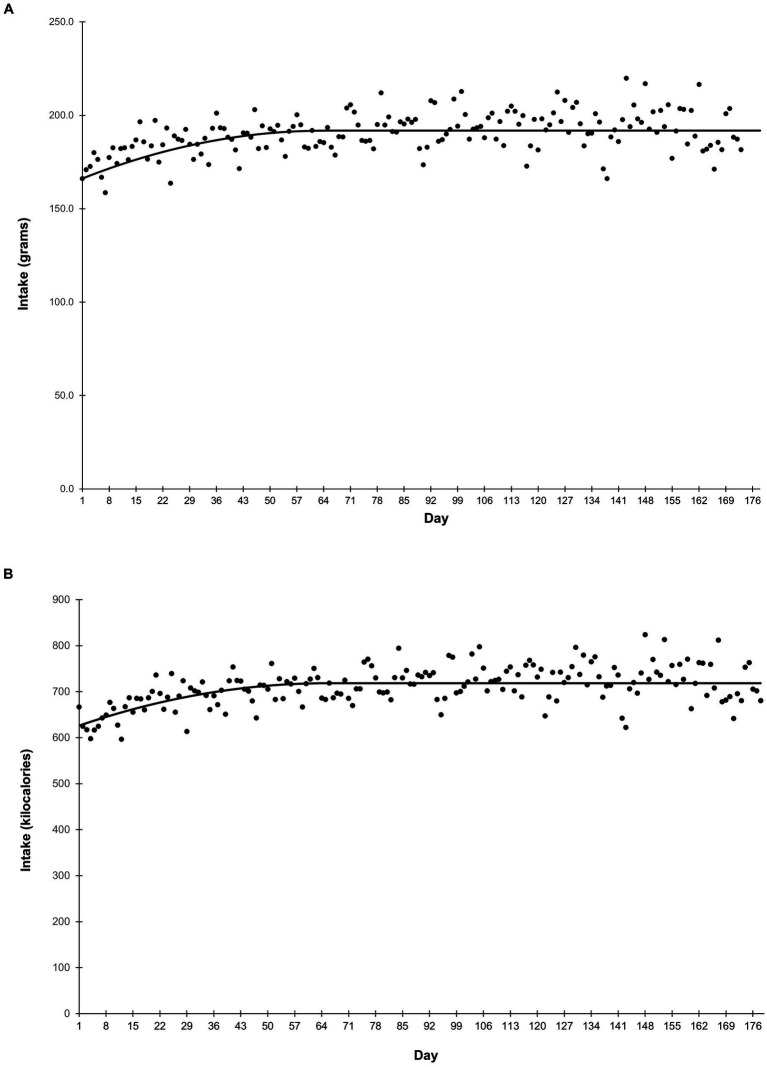
Average food intake in grams **(A)** and kilocalories **(B)** over the course of the study. Food intake in both grams and kilocalories was measured daily. Points represent the average daily intake.

**Figure 2:**
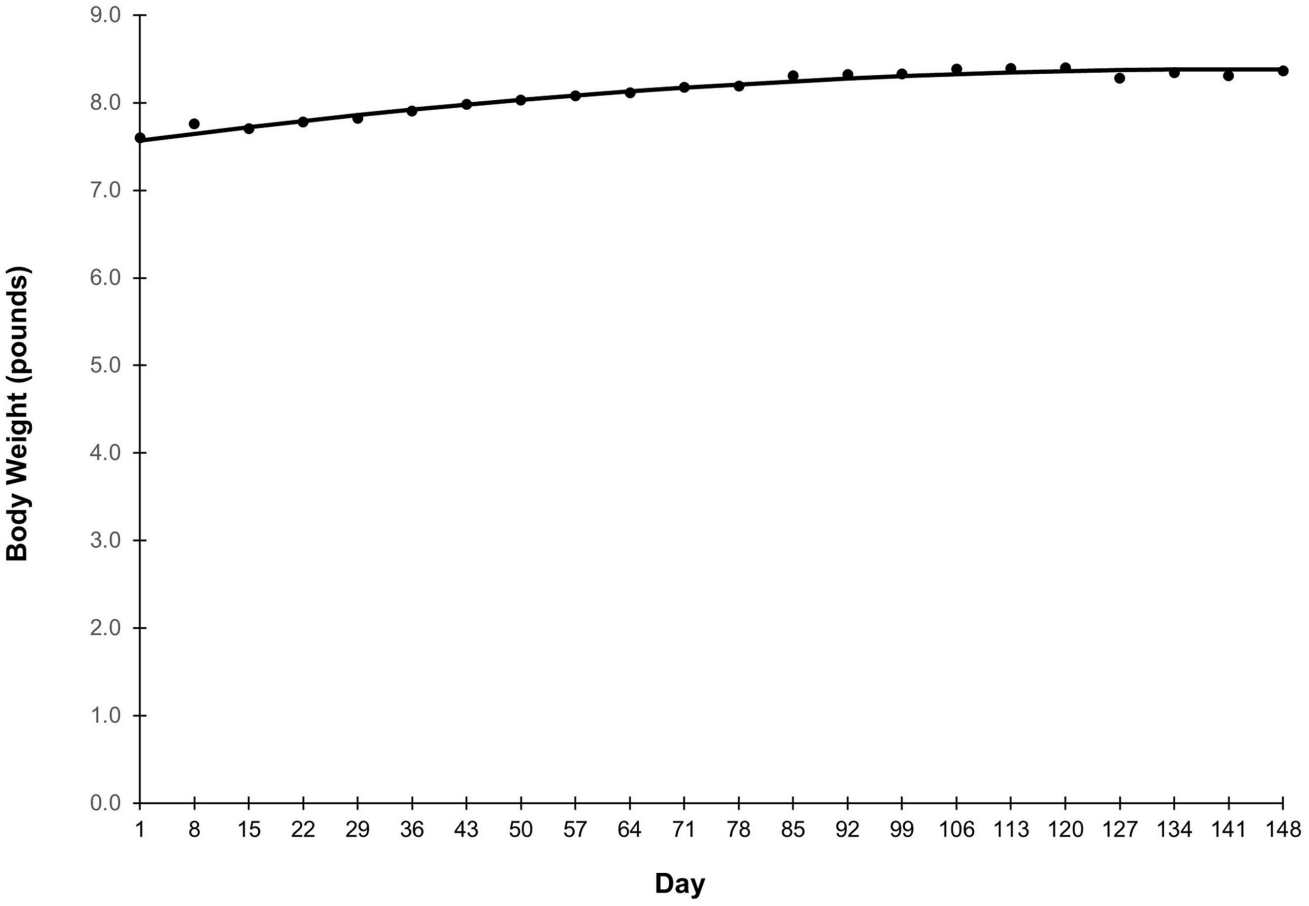
Average body weight over the study duration. Points represent the average body weight of the study population on the day specified on the *x*-axis. Body weight data from observation days after Day 148 were not included in this analysis due to small sample size.

Average food intake is shown in Figure 1. Grams intake followed a quadratic-plateau model over time (Figure 1A). There was a curvilinear increase in intake from study Days 1–64, reaching a plateau of 192 grams per day from Day 65 onward. Food intake on a calorie basis was similar (Figure 1B). Caloric intake increased from study Days 1–64, before reaching a plateau of 719 calories per day from Day 65 onward. Body weight followed a quadratic trend (*p* < 0.001) in which puppies grew rapidly at the beginning of the study, then growth slowed throughout study duration (Figure 2). Body weight data from observation days after Day 148 were not included in this analysis due to small sample size.

### 3.3 Fecal variables

**Figure 3:**
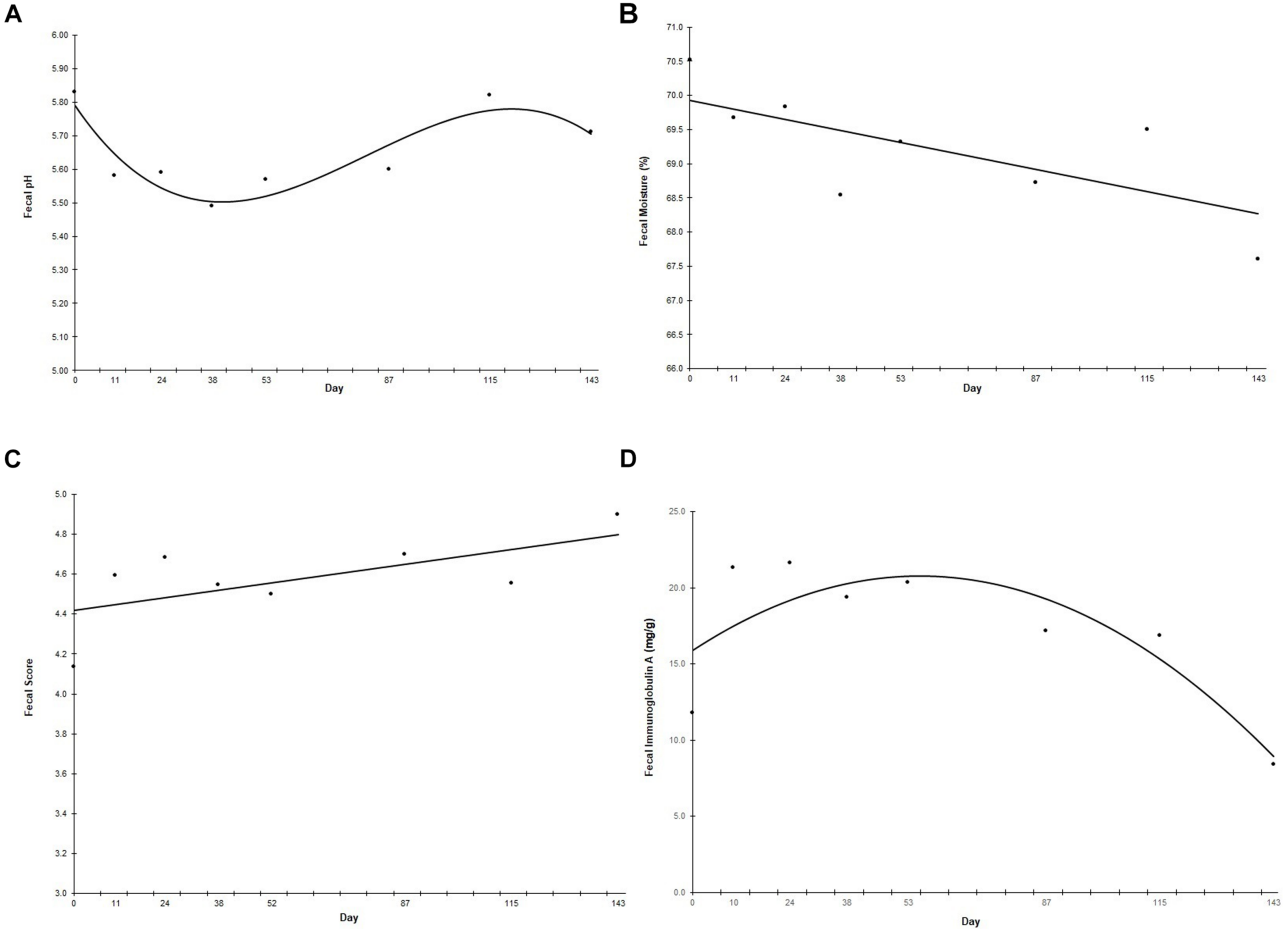
Fecal pH **(A)**, moisture **(B)**, score **(C)**, and immunoglobulin A **(D)** over the study duration. Points represent the average fecal pH, moisture, score or immunoglobulin A on fecal collection days. Fecal samples were collected every other week for the first 60 days of the study, and then once a month for the remainder of the study. “Day” on the *x*-axis reflects the test day in which fecal samples were collected, ±3 days. Fecal scores were determined using a 5-point scale, ranging from 1 (liquid diarrhea) to 5 (solid, well-formed feces at least 80% firm).

Fecal pH followed a cubic trend (*p* < 0.001) in which fecal pH decreased sharply when the animals started on the new diet until Day 38, increased from Day 38 until Day 115, and then returned to the level observed at the start of the study (Figure 3A). Fecal moisture decreased linearly (*p* = 0.041) throughout the study (Figure 3B), while fecal score increased linearly (*p* = 0.020) throughout the study (Figure 3C). There was a curvilinear trend in fecal IgA throughout the study (*p* = 0.002), in which fecal IgA increased until peaking at Day 53, then decreased to below prefeed levels (Figure 3D). Neither fecal calprotectin nor fecal ammonium changed throughout the study (data not shown).

**Figure 4:**
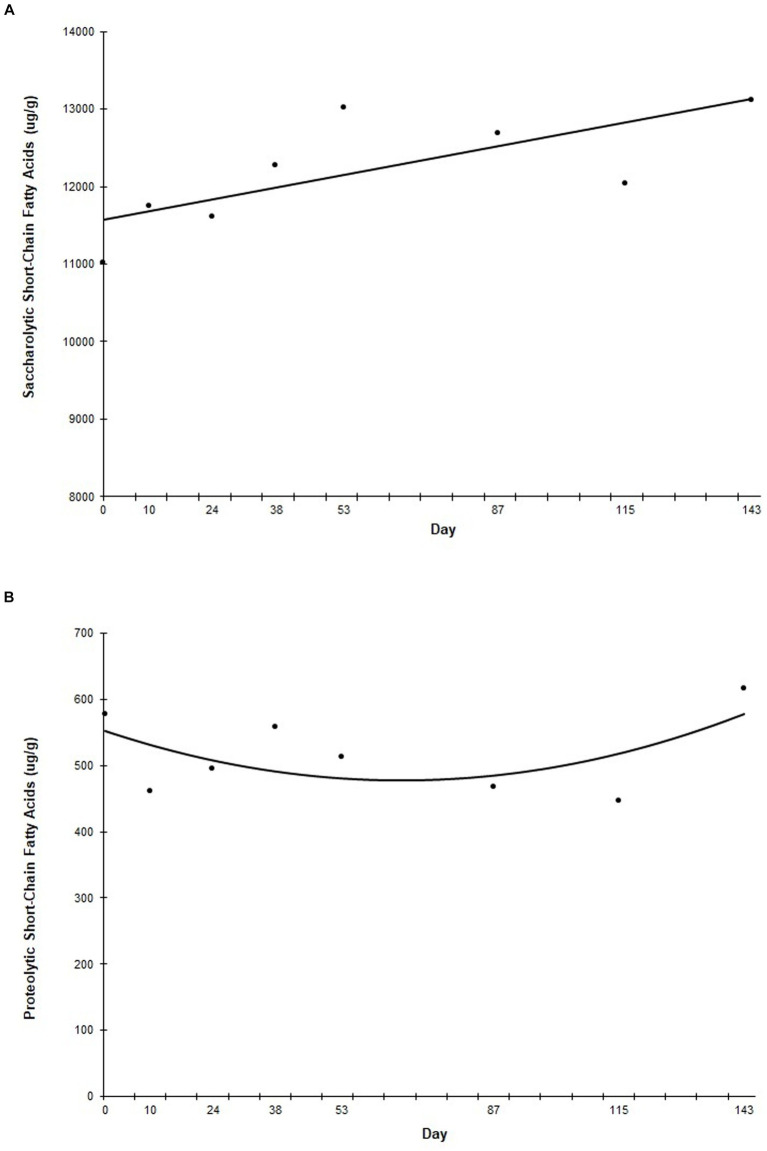
Fecal saccharolytic and proteolytic short chain fatty acid concentrations during the study. Points represent the average concentration of saccharolytic **(A)** and proteolytic **(B)** short chain fatty acids on fecal collection days. Fecal samples were collected every other week for the first 60 days of the study, and then once a month for the remainder of the study. “Day” on the *x*-axis reflects the test day in which fecal samples were collected, ±3 days.

Analysis of average fecal SCFA concentrations over the course of the study showed that as a group, saccharolytic SCFAs (including acetic acid, butyric acid, and propionic acid) increased linearly (*p* = 0.041) throughout the study (Figure 4A), while proteolytic SCFAs (including 2-methylbutyric acid, isobutyric acid, and isovaleric acid) decreased curvilinearly until Day 53, at which point they increased curvilinearly to prefeed levels (*p* = 0.040) (Figure 4B).

### 3.4 Blood variables

**Figure 5:**
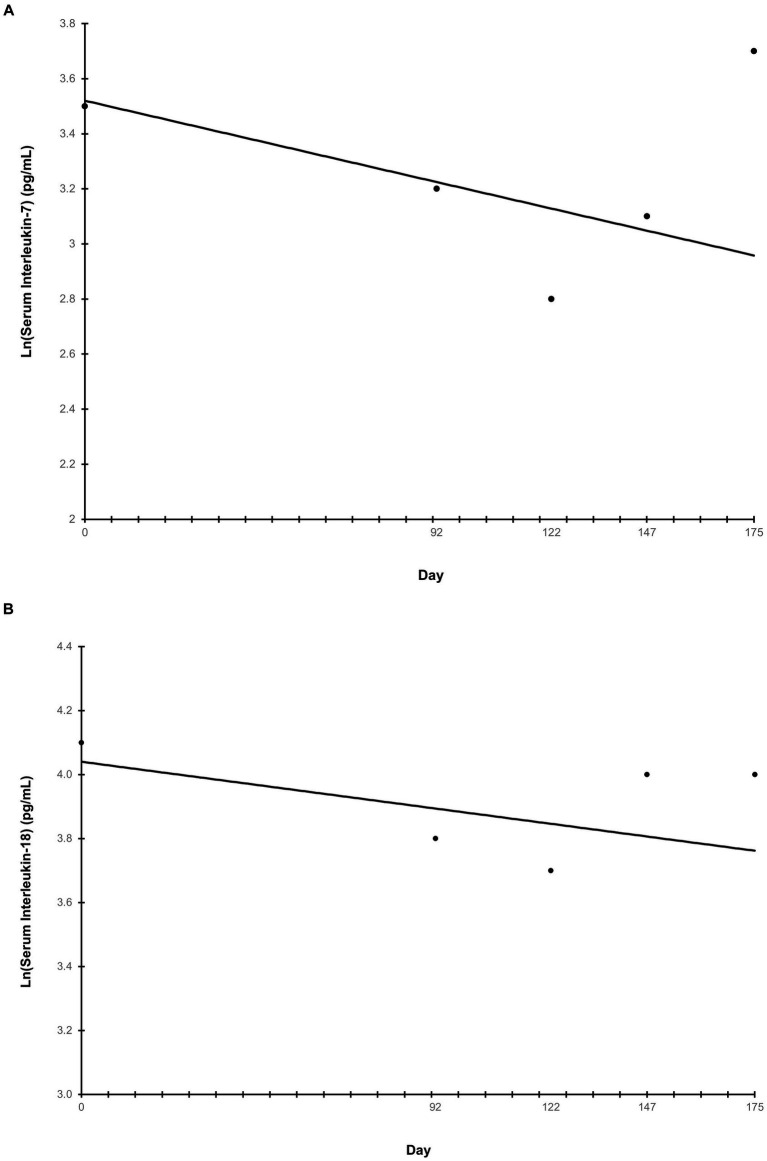
Serum inflammatory cytokines throughout the study. Points represent the average ln-transformed concentration of the cytokines interleukin-7 **(A)** and interleukin-18 **(B)** on serum collection days. Serum samples were collected at the end of the prefeed period, day 92 of consuming the test food, and upon study completion. “Day” on the *x*-axis reflects the test day in which serum samples were collected, ±3 days.

**Figure 6:**
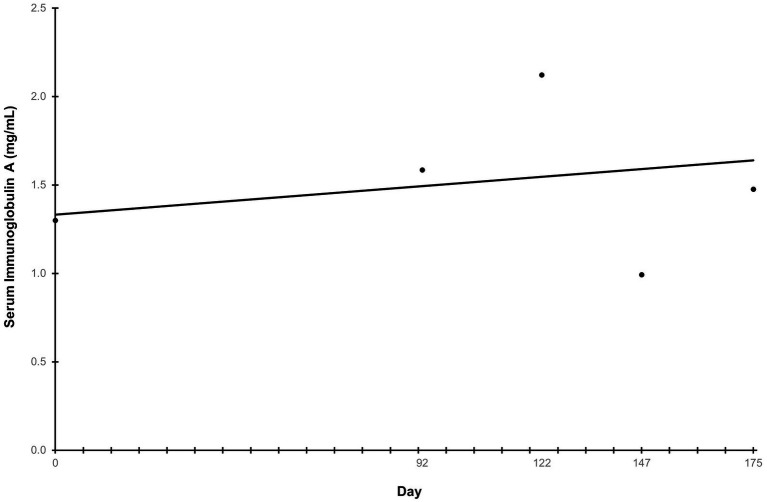
Serum immunoglobulin A concentration throughout the study. Points represent the average concentration of immunoglobulin A on serum collection days. Serum samples were collected at the end of the prefeed period, day 92 of consuming the test food, and upon study completion. “Day” on the *x*-axis reflects the test day in which serum samples were collected, ±3 days.

Ln-transformed cytokine IL-7 decreased linearly throughout the study (*p* = 0.009) (Figure 5A), while IL-18 trended towards a linear decrease (*p* = 0.062) (Figure 5B). Cytokines that had greater than 30% of measurements reported as less than the lowest level of detection, and therefore not further analyzed were interferon-gamma, interleukin (IL)-2, IL-15, IL-6, IL-10, and tumor necrosis factor alpha. The remaining cytokines that were above the limit of detection but did not exhibit any trends before or after ln-transformation were granulocyte-macrophage colony-stimulating factor (GM-CSF), IL-8, keratinocyte chemotactic-like (KC-like), interferon-gamma inducible protein 10kDa (IP-10), monocyte chemoattractant protein-1 (MCP-1) (*p* > 0.100). The average concentration of IgA in serum increased linearly throughout the study (*p* = 0.007) (Figure 6). All CBC (Table 2A) and chemistry (Table 2B) parameters were reviewed and reported as clinically normal by the attending veterinarian.

**Table 2:** Summary of complete blood count (A) and serum chemistry (B) results for each blood collection day.

Variable	Normal reference range^a^	Prefeed	Test day 92	Study completion
**(A) Blood count**				
Basophils, %	n/a	0.35 ± 0.22	0.30 ± 0.14	0.31 ± 0.16
Basophils, k/μL	0–0.04	0.032 ± 0.018	0.029 ± 0.013	0.030 ± 0.017
Eosinophils, %	n/a	2.77 ± 1.36	3.24 ± 1.78	2.86 ± 1.59
Eosinophils, k/μL	0.07–0.9	0.280 ± 0.149	0.305 ± 0.168	0.292 ± 0.204
Hemolysis, mg/dL	n/a	26.9 ± 26.8	28.1 ± 18.8	9.6 ± 3.9
Hematocrit, %	35.2–50	45.64 ± 3.62	43.80 ± 4.38	45.85 ± 2.86
Hemoglobin, g/dL	11.6–17.1	16.19 ± 1.34	15.85 ± 1.74	16.57 ± 1.16
Lymphocytes, %	n/a	30.85 ± 4.42	32.45 ± 4.97	34.66 ± 5.75
Lymphocytes, k/μL	0.75–2.45	3.046 ± 0.633	3.058 ± 0.709	3.380 ± 0.899
MCH, pg	21.4–24.5	23.70 ± 0.78	24.13 ± 0.83	23.94 ± 0.88
MCHC, g/dL	32.5–35.2	35.46 ± 0.37	36.14 ± 0.55	36.14 ± 0.51
MCV, fL	62.8–73.1	66.90 ± 1.88	66.71 ± 1.83	66.29 ± 1.84
Monocytes, %	n/a	9.01 ± 1.61	5.11 ± 0.99	4.59 ± 1.09
Monocytes, k/μL	0.13–0.7	0.887 ± 0.188	0.486 ± 0.154	0.454 ± 0.157
Neutrophils, %	n/a	57.02 ± 4.93	58.91 ± 6.16	57.59 ± 5.82
Neutrophils, k/μL	2.23–6.32	5.623 ± 0.924	5.552 ± 1.321	5.694 ± 1.726
Platelets, k/μL	137–379	287.4 ± 665.7	2710.5 ± 754.9	3132.6 ± 587.7
RBC, M/μL	5.12–7.51	6.83 ± 0.56	6.57 ± 0.63	6.92 ± 0.41
RDW, fL	31.4–36.2	32.38 ± 1.63	32.11 ± 1.41	31.72 ± 1.25
Reticulocytes hemoglobin, pg	n/a	24.99 ± 0.88	25.26 ± 1.01	25.12 ± 0.86
Reticulocytes, %	0.25–1.25	0.761 ± 0.264	0.709 ± 0.347	0.711 ± 0.335
Reticulocytes, M/μL	n/a	0.0522 ± 0.0189	0.0470 ± 0.0229	0.0489 ± 0.0229
WBC, k/μL	3.31–9.49	9.87 ± 1.39	9.43 ± 1.92	9.85 ± 2.55
**(B) Serum chemistry**				
Albumin/globulin ratio	1.1–2.4	2.20 ± 0.21	2.23 ± 0.20	2.19 ± 0.20
Albumin, g/dL	2.8–4.0	3.60 ± 0.17	3.52 ± 0.13	3.55 ± 0.19
ALP, U/L	17–134	69.5 ± 16.8	65.4 ± 23.6	57.3 ± 19.7
ALT, U/L	17–55	38.8 ± 10.9	35.9 ± 9.5	35.0 ± 7.8
Bilirubin, mg/dL	0.0–0.1	0.00 ± 0.02	0.01 ± 0.04	0.04 ± 0.05
BUN, mg/dL	7.6–19.3	14.01 ± 2.69	12.58 ± 1.74	12.88 ± 1.60
BUN/Creatinine ratio	11.3–26.4	20.28 ± 3.01	18.42 ± 2.94	18.64 ± 2.34
Calcium, mg/dL	9.0–10.8	10.40 ± 0.35	10.21 ± 0.16	10.21 ± 0.22
Chloride, mmol/L	108–116	111.25 ± 1.66	112.51 ± 1.51	113.67 ± 1.73
Cholesterol, mg/dL	127–318	163.3 ± 30.1	180.3 ± 42.3	182.2 ± 34.0
Creatinine, mg/dL	0.5–1	0.691 ± 0.085	0.692 ± 0.100	0.695 ± 0.071
Globulin, g/dL	1.5–2.6	1.65 ± 0.16	1.59 ± 0.12	1.64 ± 0.15
Glucose, mg/dL	79–116	105.3 ± 7.8	100.5 ± 7.4	102.6 ± 7.9
Icteric	n/a	0	0	0
IPF, %	n/a	4.37 ± 6.36	5.83 ± 8.94	2.66 ± 4.08
IRF, %	n/a	22.03 ± 6.91	20.24 ± 7.78	21.17 ± 7.02
Lipemic, mg/dL	n/a	5.09 ± 6.34	2.77 ± 1.51	6.00 ± 1.86
Magnesium, mg/dL	1.7–2.2	1.75 ± 0.08	1.80 ± 0.10	1.87 ± 0.09
Na/K ratio	29–40	32.41 ± 2.24	30.327 ± 2.07	31.45 ± 1.82
Phosphorus, mg/dL	2.3–4.7	4.70 ± 0.65	4.38 ± 0.64	4.32 ± 0.52
Potassium, mmol/L	3.7–5.1	4.508 ± 0.314	4.851 ± 0.315	4.746 ± 0.248
ProteinT, g/dL	4.8–6.1	5.25 ± 0.26	5.11 ± 0.17	5.19 ± 0.28
Sodium, mmol/dL	145–150	145.32 ± 1.32	146.00 ± 1.45	149.10 ± 1.33
Triglycerides, mg/dL	19–119	39.8 ± 11.9	37.1 ± 13.2	37.7 ± 16.3

ALP, alkaline phosphatase; ALT, alanine transaminase; BUN, blood urea nitrogen; CBC, complete blood count; IPF, immature platelet fraction; IRF, immature reticulocyte fraction; MCH, mean corpuscular hemoglobin; MCHC, mean corpuscular hemoglobin concentration; MCV, mean corpuscular volume; n/a, not applicable; RBC, red blood cells; RDW, red cell distribution width; SD, standard deviation; WBC, white blood cells.

a The normal reference range is based on internal definitions determined by Hill’s Pet Nutrition Center veterinarians. Blood samples were collected upon the end of the prefeed period, on day 92 of consuming test food, and upon study completion. Data is presented as mean ± SD.

## 4 Discussion

This study was conducted to help elucidate the effects of prebiotics in growing animals, where knowledge is currently limited. In this study, we evaluated the growth and gastrointestinal health of dogs between 8 and 14 months of age fed a novel prebiotic fiber blend, a source of soluble and insoluble fiber as well as fiber-bound polyphenols. Puppies fed a test food that contained this novel prebiotic blend grew as expected for puppies of this age (32). Lower food intake was observed at the beginning of the test period, but then increased as appropriate for their age in correspondence with their increased body weight (3). Both CBC and serum chemistry analyses were considered clinically normal for all dogs. Therefore, puppies remained in good health while consuming the test food fortified with this novel prebiotic fiber blend.

Fecal moisture decreased linearly and fecal score increased linearly throughout the treatment period, suggesting improved GI health and stool quality throughout the study. This aligns with previous work in adult dogs showing that food fortified with the prebiotic fiber blend has beneficial effects on stool quality, both over time and in comparison to dogs fed a control food without the fiber blend (24, 25, 27). The decline in fecal moisture in the puppies throughout the study duration is also indicative of reduced fecal water loss, which indicates a potential association between the test food and body water retention. In addition, fecal pH was consistently below 6. In humans, a slightly acidic pH has been shown to help prevent growth of pathogenic bacteria in the gut, support additional fermentation and production of beneficial microbial metabolites, and support other areas of whole-body health. Therefore, a slightly acidic pH may be indicative of a more favorable GI environment (33). Previous studies have shown that the addition of the prebiotic fiber blend decreased fecal pH over time in healthy adults dogs and adult dogs with enteritis/gastroenteritis (27). A linear decrease in pH over time was not seen in this present study with puppies, though fecal pH did change cubically before ending at a lower pH than baseline. The baseline fecal pH of the puppies was similar to the final pH reported in the study of adult dogs, and thus, this value may indicate a healthy environment favoring microbial saccharolysis in the gut.

As a group, the saccharolytic SCFAs increased linearly throughout the study. Saccharolytic SCFAs are important for maintaining intestinal homeostasis, providing fuel for intestinal epithelial cells, and strengthening the gut barrier function, and reduced levels of saccharolytic SCFAs have been associated with diseases, such as irritable bowel syndrome (34). In contrast, putrefactive SCFAs had a curvilinear trend and were lower than baseline at one time point towards the end of the study. Putrefactive SCFAs, also known as branched chain fatty acids (BCFAs) are the products of proteolytic fermentation by gut microbes. An increase in BCFA production has been associated with higher levels of harmful compounds, including uremic toxins (27). Previous studies have found a decrease in BCFAs in dogs with chronic large bowel diarrhea and an increase in some individual SCFAs in both healthy adult dogs and adult dogs with chronic large bowel diarrhea when fed a food fortified with this prebiotic fiber blend (24, 25, 27). The results of this present study are aligned with findings seen in adult dogs, and indicate a shift in the gut environment of puppies fed this novel prebiotic towards one that is more favorable for saccharolytic fermentation, which is associated with favorable GI function and overall health (27, 34).

Immunoglobulin A is abundant in mucosal tissue, particularly the GI tract, and has been found to be important in maintaining a healthy GI microbiome (35). A steady increase in serum IgA over the course of the study may indicate an improved immune and/or anti-inflammatory response throughout study duration (36). No trends were observed in fecal calprotectin or ammonium concentrations during the study. The absence of changes in these fecal parameters may indicate the maintenance of a healthy GI environment in these growing dogs fed the test food with the novel prebiotic fiber (16, 37–39). A more in-depth analysis of the effects of the fiber blend on the GI microbiota of puppies as part of this study is currently being conducted.

Of the inflammatory cytokines analyzed for changes over time, IL-7 decreased linearly while IL-18 trended towards linear decrease, which may indicate a reduction in inflammation throughout the study. An increased level of serum IL-7 is associated with inflammatory diseases, like inflammatory bowel disease and rheumatoid arthritis in humans (40), while IL-18 has been associated with intestinal inflammation in mice (41–43). Previous research in humans has shown that after two years of consuming a Mediterranean diet, which consists of foods high in monounsaturated fat, polyunsaturated fat, and fiber, patients had significantly reduced levels of serum IL-7 and IL-18, and lower levels than patients in the control group who did not consume the Mediterranean diet (44). Additionally, an increased dietary fiber intake is associated with a reduction in pro-inflammatory cytokines, including IL-18, in both diabetic and nondiabetic subjects (45). In dogs, restricted feeding of a high-protein, high-fiber food resulted in weight loss and a corresponding decrease in inflammatory cytokines (46), but more work is needed to evaluate the effects of fiber consumption on inflammatory markers in dogs specifically. Nevertheless, foods containing this novel fiber blend may help in modulating inflammation in dogs, though more work is needed to confirm this hypothesis. Those cytokines with less than 30% of values below the lower limit of detection were detected in the serum of almost all puppies during each serum collection. Therefore, we conclude that some cytokines tend to be present at detectable levels in young dogs while other cytokines may frequently be present below detectable levels.

Limitations of this study include that it was conducted in puppies 8 months of age and older. Although the use of a prebiotic in younger puppies is of significant interest, because this is the first study of this novel prebiotic fiber blend in puppies, the authors concluded that an older population would be more homogeneous, with less risk of comorbid conditions that could have affected the results. A study in puppies younger than 8 months of age is under consideration. Another limitation is that parameters were not compared between animals fed and not fed this novel prebiotic (i.e., a control group). However, the data were analyzed for clinically meaningful changes over time starting with baseline, which is when the puppies were fed a similar, commercially available food but without the prebiotic fiber blend. These tests for trends over time in the same population removed animal-to-animal variation. Nevertheless, a randomized clinical trial with a group consuming the test food and a control group may be an area for future investigation. It should also be noted that the objective of this study was to evaluate the benefits of a food fortified with the novel prebiotic fiber blend on the growth, development, and GI health of puppies, and not as a strategy for managing a health condition. Along with the previously explained benefits, as expected, there were no clinically relevant negative effects. As this is one of the first studies investigating the effects of a food supplemented with a prebiotic blend in puppies, this present study provides valuable information for developing foods that provide optimal nutrition for growing companion animals.

## 5 Conclusion

This study reveals that puppies continue to grow and develop normally and exhibit characteristics indicative of good GI health, including improved stool quality, reduced fecal moisture, and increased SCFAs, when fed a growth food fortified with a novel prebiotic fiber blend for up to 175 days. The benefits observed in puppies in this study align with those observed in adult dogs fed a food containing this novel fiber blend, indicating that the developing GI tract responded similarly to that of healthy adult dogs. Thus, feeding puppies a blend of soluble and insoluble fibers may serve as a nutritional strategy to support GI health, growth and development, and overall well-being.

## Data availability statement

The raw data supporting the conclusions of this article will be made available by the authors, without undue reservation.

## Ethics statement

The animal study was approved by Hill’s Pet Nutrition Institutional Animal Care and Use Committee (IACUC). The study was conducted in accordance with the local legislation and institutional requirements.

## Author contributions

AM: Investigation, Data curation, Writing – review & editing, Writing – original draft. LM: Writing – review & editing, Writing – original draft, Supervision. JB: Writing – review & editing, Writing – original draft, Formal analysis. LH: Supervision, Writing – review & editing, Writing – original draft.
